# Keele Aches and Pains Study protocol: validity, acceptability, and feasibility of the Keele STarT MSK tool for subgrouping musculoskeletal patients in primary care

**DOI:** 10.2147/JPR.S116614

**Published:** 2016-10-14

**Authors:** Paul Campbell, Jonathan C Hill, Joanne Protheroe, Ebenezer K Afolabi, Martyn Lewis, Ruth Beardmore, Elaine M Hay, Christian D Mallen, Bernadette Bartlam, Benjamin Saunders, Danielle A van der Windt, Sue Jowett, Nadine E Foster, Kate M Dunn

**Affiliations:** 1 Arthritis Research UK Primary Care Centre, Research Institute of Primary Care and Health Sciences, Keele University, Keele; 2 Health Economics Unit, University of Birmingham, Birmingham, UK

**Keywords:** musculoskeletal, stratified care, pain, predictive, risk, primary care

## Abstract

Musculoskeletal conditions represent a considerable burden worldwide, and are predominantly managed in primary care. Evidence suggests that many musculoskeletal conditions share similar prognostic factors. Systematically assessing patient’s prognosis and matching treatments based on prognostic subgroups (stratified care) has been shown to be both clinically effective and cost-effective. This study (Keele Aches and Pains Study) aims to refine and examine the validity of a brief questionnaire (Keele STarT MSK tool) designed to enable risk stratification of primary care patients with the five most common musculoskeletal pain presentations. We also describe the subgroups of patients, and explore the acceptability and feasibility of using the tool and how the tool is best implemented in clinical practice. The study design is mixed methods: a prospective, quantitative observational cohort study with a linked qualitative focus group and interview study. Patients who have consulted their GP or health care practitioner about a relevant musculoskeletal condition will be recruited from general practice. Participating patients will complete a baseline questionnaire (shortly after consultation), plus questionnaires 2 and 6 months later. A subsample of patients, along with participating GPs and health care practitioners, will be invited to take part in qualitative focus groups and interviews. The Keele STarT MSK tool will be refined based on face, discriminant, construct, and predictive validity at baseline and 2 months, and validated using data from 6-month follow-up. Patient and clinician perspectives about using the tool will be explored. This study will provide a validated prognostic tool (Keele STarT MSK) with established cutoff points to stratify patients with the five most common musculoskeletal presentations into low-, medium-, and high-risk subgroups. The qualitative analysis of patient and health care perspectives will inform practitioners on how to embed the tool into clinical practice using established general practice IT systems and clinician-support packages.

## Background

Musculoskeletal conditions represent a considerable burden worldwide. The Global Burden of Disease study findings show that such conditions as low-back pain are the leading cause of years lived with disability in Western Europe and Australia, and that musculoskeletal conditions overall account for 6.8% of global disability-adjusted life-years, comparable to cancer (7.8%), ischemic heart disease (5.2%), and mental disorders (7.4%).^1^ This burden is reflected in health care use: musculoskeletal consultations account for around a fifth of all consultations in UK primary care.^2^,^3^ Prevalence of persistent musculoskeletal pain is high, estimated at 25%–32%,^4^ and recurrence is common.^5^ Musculoskeletal conditions thus have a major impact on the individual, health care, and society.^6^

While regional musculoskeletal pain presentations (ie, pain specific to a certain body region) are recognized by virtue of the anatomical location (eg, back, neck, shoulder, knee) and associated features of such pain presentations (ie, impact on physical function), there is strong substantive evidence that pain presentations share common underlying mechanisms.^7^ Co-occurrence of pain in different body regions is high,^8^ and risk of poor outcome is increased for those with multisite pain.^9^,^10^ Additionally, patients with different regional musculoskeletal pains (eg, back, neck, shoulder, or knee pain) and those with multisite pain share similar prognostic factors.^11^–^13^ Previous work has demonstrated that a chronic pain-risk score, incorporating prognostic indicators, and developed in patients with back pain,^14^ is valid for use among patients with pain at different anatomical sites, and in different settings.^15^–^17^ Other work^18^ has shown that patients can be screened to assess the presence of prognostic factors, irrespective of the specific location of the musculoskeletal condition. However, previous methods and tools, such as the Örebro Musculoskeletal Pain Screening Questionnaire were not designed for use specifically within primary care^19^,^20^ or to stratify patients based on their level of risk and recommend matched treatments (stratified care). One model of stratified care is to create subgroups based on a prognostic profile.^21^ This approach of stratified care can “fast-track” patients to the appropriate treatment by supporting clinical decision making, and has the potential to maximize treatment benefit, reduce harm, and increase health care efficiency.^22^

One prognostic tool (the STarT [subgroups for targeted treatment] Back tool) was successfully developed for low-back pain patients in primary care,^23^ specifically to assist in matching patients to different treatments. The tool consists of nine items, and utilizes cutoff point scores to identify three prognostic subgroups (low, medium, and high risk of persistent back-related physical disability). All physical and psychological prognostic indicators in the STarT Back tool were chosen based on their potential to be modifiable through treatment. Subsequent use of the tool, as part of a stratified care model in which subgroups of patients were matched to different treatments, has demonstrated superior clinical and economic outcomes compared to best current practice and usual primary care.^24^,^25^

Given this promising research, the predictive ability of a modified, generic version of the STarT Back tool was tested in a broader range of patients with musculoskeletal pain presentations (neck pain, upper-limb pain, lower-limb pain, multisite pain, and back pain). The predictive performance of this new draft – “Keele STarT MSK tool” – was assessed in secondary analysis of two large data sets of patients with musculoskeletal pain conditions consulting physiotherapy services (PhysioDirect study)^26^ and musculoskeletal primary–secondary care-interface services (SAMBA study).^27^ Results showed a moderate-to-good level of predictive ability of the draft tool in the identification of patients who developed persistent disabling pain, eg, prediction of poor outcome using area under the curve (AUC) – back pain AUC 0.72, 0.79, neck pain AUC 0.82, 0.88, upper-limb AUC 0.79, 0.86, lower-limb AUC 0.79, 0.86, and AUC 0.83, 0.82 multisite pain – in PhysioDirect and SAMBA, respectively. However the results also indicated that different optimal cutoff points were required for different regional pain presentations (eg, lower limb, upper limb, spinal pain). Subsequent informal discussions on the applicability of the draft tool were carried out with GPs and first-contact health care practitioners (HCPs). Feedback from discussions indicated that due to the complexities of scoring the draft tool (ie, different cutoff points per body region) added to the current time pressures within primary care, a single generic tool with one set of cutoff points would be preferable in clinical practice. Furthermore, a more straightforward tool would facilitate links to matched treatments within a stratified care model.

The overall aims of the Keele Aches and Pains Study (KAPS) are further to refine and validate the draft Keele STarT MSK tool, and based on our previous experience in implementing stratified primary care for patients with low-back pain^25^ to collect information about the anticipated acceptability and feasibility of using such a tool, as well as identify barriers to implementation and practical solutions to these barriers.^28^ We aim to develop the tool for use with patients who have the five most common musculoskeletal pain presentations in primary care: back, neck, shoulder, knee, or multisite pain.^3^ The KAPS forms part of a larger National Institute for Health Research (NIHR) program of four linked work packages. The overall objective is to stratify patients with a range of musculoskeletal presentations based on risk of poor outcome, identify treatment options that are matched to each stratum, test the acceptability of this model in primary care, test whether such an approach is effective in the improvement of patient outcomes and experience, and test whether it is cost-effective.

### Aims and objectives

Within the context of primary care and the five most common musculoskeletal presentations, there are two key aims of the KAPS: 1) to refine and externally validate the draft Keele STarT MSK tool, and 2) to explore how such a tool might best be implemented within clinical practice. Specific objectives include:

1. refine the Keele STarT MSK tool based on face, discriminant, construct, and predictive validity
2. determine the tool risk-strata cutoff points based on optimal predictive values and suitability for matched treatment options
3. estimate the proportions of patients classified at low, medium, and high risk of poor outcome and describe their characteristics, including patient health care utilization and costs and health-related quality of life
4. examine the validity of a refined tool (predictive, external validation)
5. explore clinicians’ perspectives on the acceptability and “added value” of stratified care based on prognosis compared to current primary care management of patients
6. explore patients’ perspectives about stratified primary care based on prognosis (eg, patient subgrouping using the tool, matched treatment options)
7. identify clinicians’ and patients’ perceived barriers and solutions to using the draft Keele STarT MSK tool and matched treatment options in clinical practice.

## Methods/design

Ethical approval for this study has been granted by the South East Scotland Research Ethics Committee in the UK (14/SS/0083).

### Study design and setting

This will be a prospective observational cohort study with a linked qualitative study within a primary care setting.

### Participating patients

Consecutive adult patients who visit their GP with one (or more) of the five most common musculoskeletal pain presentations – back, neck, shoulder, knee, or multisite pain – will be invited to participate in the cohort study. A subsample of patients who respond and agree to participate in the cohort study will be invited to take part in the linked qualitative focus groups and interviews. In addition, GPs and other HCPs involved in musculoskeletal treatments (eg, physiotherapists) will be invited from the participating general practices and related services, or known clinical networks, to take part in focus groups and interviews.

### Inclusion/exclusion criteria

Inclusion criteria are patients registered at participating general practices, aged 18 years or over, consulting with the included musculoskeletal pain presentations, and able to read and understand English. All consecutive patients will be invited to participate, regardless of prior consultations. Exclusion criteria are indication of serious pathology (eg, suspected fracture, cancer), inflammatory arthritis, crystal disease, spondyloarthropathy, polymyalgia rheumatica, pregnancy-related pain problems, urgent cases (eg, cauda equina syndrome), or vulnerable patients (eg, experienced recent trauma, cognitive impairment, dementia, or terminal illness). There are no inclusion/exclusion criteria for the GP and other HCP focus groups and interviews, but researchers will attempt to recruit to maximize sample variation on the basis of age, sex, clinical experience, location, and clinical specialty. All focus groups will be conducted in English.

### Cohort study recruitment

Individual patients who consult with back, neck, shoulder, knee, or multisite pain will be identified through relevant musculoskeletal symptom and diagnostic Read codes. The Read codes have been previously used by Jordan et al,^3^ with additional amendments and checks for relevance and completeness carried out by GPs in the research team. GP practice staff and Clinical Research Network staff will support weekly-to-fortnightly electronic record searches for the relevant Read codes at participating primary care practices to identify potentially eligible patients. Patients’ GPs will be invited to screen patient lists prior to mailing. Eligible patients will receive an invitation letter and survey pack (participant information sheet, baseline questionnaire, and prepaid return envelope) from their GP shortly after their musculoskeletal pain consultation (index consultation).

Return of the completed questionnaire by the patient will signify consent to participate in the cohort study (ie, consent to receive further questionnaires), as specified within the participant information sheet. In addition, specific consent will be requested to allow access to patients’ medical records and to be contacted about further linked research (ie, linked qualitative focus groups and interviews). Baseline nonresponders will be sent a reminder postcard after 2 weeks and a reminder letter (with a copy of the baseline questionnaire) 2 weeks after that. Patients who indicate that they do not wish to take part at any point will not be sent any further correspondence. Patients will not receive any payment or financial incentive to take part.

### Cohort study follow-up

**Figure 1:**
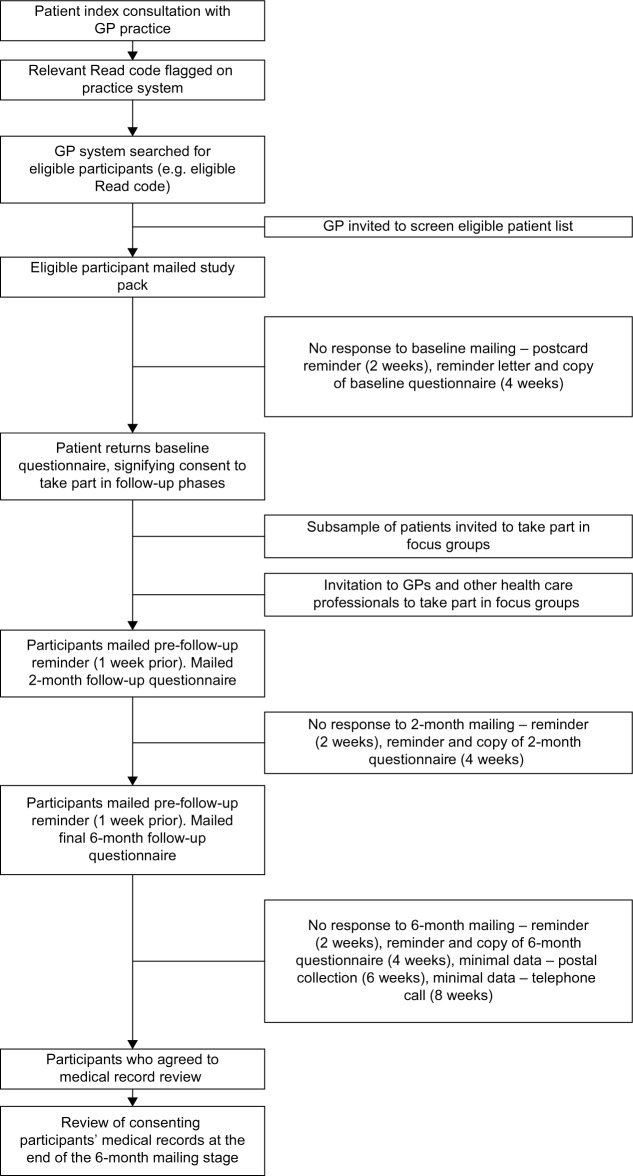
Flowchart of recruitment procedure for the Keele Aches and Pains Study.

All patients who have signified consent to take part will be sent follow-up questionnaires 2 months and 6 months after baseline. Wording on each follow-up questionnaire will be personalized to indicate which anatomical area they originally consulted about during their index consultation (ascertained from patients’ baseline questionnaire responses). One week before follow-up questionnaire mailing, patients will receive a reminder (pre-follow-up) that they are about to receive their next questionnaire. After the 2-month and 6-month questionnaire mailing, nonresponders will receive a reminder after 2 weeks (postcard, text message, or email), and a repeat questionnaire 2 weeks later. After the final 6-month mailing, a brief questionnaire containing only the key-outcome measures (minimal data collection [questionnaire]) will be sent at 6 weeks to nonresponders. Finally, telephone contact will be attempted at 8 weeks for those who have provided a telephone number on their baseline questionnaire, to collect key-outcome data (minimal data collection [telephone]). Telephone calls will be carried out by a research nurse. Please see Figure 1 for a flowchart of the recruitment stages for the KAPS.

The recruitment strategies outlined follow a recent Cochrane review of best practice to increase response to postal questionnaires,^29^ and follow guidelines from the World Health Organization for research to adapt to the rapid changes in the way people communicate (eg, mobile technologies, telephone communication, Internet).^30^

### Cohort study data collection

The draft Keele STarT MSK tool and the key primary quantitative measures will be included in the questionnaire measures at all time points. The key primary quantitative measures are physical health (short-form [SF]-36 version 2 physical component summary score),^31^,^32^ pain-intensity (least, average, and current pain),^33^ and pain interference (Promis pain-interference scale).^34^ The study will also include a measure of health-related quality of life (EuroQol [EQ]-5D-5L)^35^ at all time points. The minimal data-collection questionnaire will include the SF-12, which is a shortened version of the SF-36 version 2.^36^ Secondary measures include aspects of pain duration, spread and bother, self-reported change in symptoms (follow-up stages only), pain catastrophizing, pain self-efficacy, illness perceptions, sleep problems, physical activity, social support, health literacy, comorbidity, employment status, and factors about employment if applicable (see Table 1 for full list of measures, references, and time points for data collection). There will also be questions in the follow-up questionnaires on previous National Health Service (NHS) and non-NHS health care utilization (eg, contact with GP, nurse, physiotherapist, osteopath, and use of X-rays, surgery, prescriptions, over-the-counter medication). In addition, single-item questions derived and adapted from the established measures outlined will be included. The selection of these items is based on their prognostic performance in primary care musculoskeletal pain studies.^37^–^40^

### Cohort study medical record review

For those who give consent, analysis of medical records will supplement patient self-report information (eg, additional information on prescriptions, consultation frequency, referral for further treatment, diagnostic tests, and sickness certification/fit notes). The time scale for medical record review is 1 month prior to the patient’s index consultation, up to 6 months after their index consultation.

### Cohort study sample size

Based on a 6-month recruitment period at each participating practice, from a source population of approximately 40,000 registered adult patients with an estimated annual consultation prevalence of 20% for musculoskeletal consultations,^3^ we estimate that there will be approximately 3,000 eligible patients who consult with the five most common musculoskeletal pain presentations during the recruitment period. With an estimated response of 60%–70% (based on previous studies with similar populations),^33^,^41^ we anticipate recruiting 1,800 patients at baseline and retaining 1,250 patients at 6-month follow-up. Calculations show a minimum of 100 patients per outcome category (risk subgroup) is required for external validation.^42^ Based on our previous studies,^23^–^26^ we anticipate that at least 10% of musculoskeletal patients in primary care will be classified at high risk of poor outcome (the smallest risk stratum), and thus each risk subgroup will include at least 125 follow-up respondents, providing adequate power for validation of the draft Keele STarT MSK tool using data from 6-month follow-up.

### Cohort study analysis

Refining the Keele STarT MSK tool and evaluating its validity will be carried out in four stages. First, the performance of the draft tool will be described. Patients will be classified, using the draft tool (including cutoff points based on the original STarT Back tool, ie, total score <4, “low risk”; total score ≥4 and psychosocial subscale score <4, “medium risk”; total score ≥ 4 and psychosocial-subscale score ≥4, “high risk”), as being at low, medium, or high risk of poor outcome.^23^ They will be described in terms of their baseline characteristics: pain (pain intensity, pain interference, pain bother), physical function (SF-36 version 2 physical component score [PCS]), mental health (SF-36 version 2 mental component score), general health (SF-36 version 2, sleep problems, fatigue, physical activity, comorbidity), psychological reactions to pain (coping strategies, pain self-efficacy, illness perceptions), quality of life (EQ-5D-5L), health literacy (single-item literacy screener), and social factors (eg, available emotional and instrumental social support, employment status, socioeconomic status). This will allow assessment of the draft tool’s discriminant validity, ie, ability to discriminate between low-, medium-, and high risk subgroups. Hypotheses will be tested based on the assumption that participants in medium- and high-risk subgroups will have higher pain intensity,^54^ more pain bother, poorer physical function, and lower levels of physical and mental health. Evaluation will be based on analysis of variance using linear constraints for numerical measures and the *χ*^2^ test for trends for comparing and testing associations in categorical outcome measures. In order to assess construct validity, risk subgrouping based on the draft tool will be compared with classification based on the Örebro Musculoskeletal Pain Screening Questionnaire,^20^ an existing tool for the prognostic classification of patients with musculoskeletal conditions (medium and high scores will be combined for purposes of this comparison). In the subsample of participants with low-back pain, comparisons will also be made with risk classification according to the STarT Back tool. The results will be expressed as percentage agreement and weighted *k*-values with 95% confidence intervals. Test of linear association will also be given by the *χ*^2^ test for trend.

The predictive performance of the draft Keele STarT MSK tool will be investigated by assessing the ability of the tool to predict outcome at the 2-month follow-up. It is hypothesized that participants with lower baseline scores (ie, those at “low” risk (or “medium” risk compared to “high” risk) will have better outcomes. Evaluation will be based on analysis of variance using linear constraints for numerical 2-month measures, and the *χ*^2^ test for trends for comparing and testing associations in categorical 2-month outcome measures. In relation to the key outcomes of physical health (SF-36-PCS), predictive performance of the draft tool will be expressed in terms of percentage of variance explained (parametric and nonparametric Nagelkerke *R*^2^), calibration (calibration slope and Hosmer–Lemeshow test), and area under the receiver-operating characteristic curve (*C*-statistic) via receiver-operating-characteristic analysis for discrimination, including dichotomies for the SF-36-PCS based on lower tertiles extracted from a similar cohort,^26^ ie, 37.17 and 39.61 at 2- and 6-month follow-up, respectively.^55^,^56^ A replication of this analysis using the SF-36-PCS will be carried out for pain intensity (eg, a score of 5 or more denotes moderate/severe pain).^57^ In addition, as a sensitivity analysis, variation in performance will be examined across participants with different index pain sites.

Second, opportunities to refine and improve upon the draft Keele STarT MSK tool will be investigated. In particular, face and content validity, completion rates, and predictive performance of the tool will be optimized by the inclusion of additional or alternative items or the removal of items. Predictive performance will be assessed against physical health (SF-36-PCS) and pain-intensity scores at 2-month follow-up. Primary evaluation will use the whole sample, but sensitivity analyses will be carried out for participants with pain at each index site. A number of single-candidate item questions will be included in baseline questionnaires (physical, psychological, or social factors); these are based on an updated review of prognostic factors for musculoskeletal pain in primary care, overview of other comparable tools (Örebro and Von Korff risk score), and examination of other questions used to measure the same and/or other identified key domains. As with the development of the Keele STarT Back tool,^23^ all candidate items will be potentially modifiable by treatment. This analysis will be carried out in stages by substituting, adding, or removing one item at a time. Decisions about whether to change, add, or remove an item will be based on all available information (face and content validity, item-completion rate, and predictive performance overall and across pain sites). For example, if swapping one item in the draft tool for a new candidate item with better face validity leads to similar item-completion rates and improves predictive performance compared to the draft tool, a decision would be made to remove the existing item and add the new item.

The refined tool will then be investigated to see whether it is exhaustive by checking whether items reflecting different domains can be added to improve predictive validity substantially. In addition, the potential for item redundancy will be examined by reviewing the tool’s predictive performance versus simplification by exclusion of single items one at a time. At all stages, the main focus will be on improved predictive performance across the total study population, but review of predictive validity will also be extended to individual index pain sites, and face and construct validity will be taken into account.

The third step is to identify tool risk-strata cutoff points. Two approaches will be considered. One approach will identify the optimal cutoff points on the 0–9 scale that stratifies the population into three subgroups of low, medium, and high risk based on evaluation of sensitivity/specificity, predictive values (positive/negative), and likelihood ratios (positive/negative) against clinical thresholds for the SF-36-PCS and pain, as detailed earlier. An alternative method used in the development of the STarT Back tool^23^ is based on a separate cutoff point using a psychosocial subscale to identify complex patients. In this approach, items that could be classified as key in identifying patients who are at high risk (ie, psychosocial, complex problems) will be identified. These two approaches to defining cutoff points will be compared in terms of discriminant, construct, and predictive validity (as described in steps 1 and 2) and reviewed by the study team for feasibility and fit.

The fourth and final stage is to determine whether the refined version of the tool has external validity. This will be done by assessing the discriminant, construct, and predictive validity of the refined tool against 6-month outcomes. In addition, these same analyses will be conducted following multiple imputation of missing data to assess robustness of the tool’s properties. At all stages of the analysis, a clinical advisory group and user representatives (patient and public involvement of people with similar musculoskeletal conditions) will be consulted about the draft versions of the Keele STarT MSK tool, and any suggested changes will be discussed, to ensure acceptability, face validity, and clinical utility of the final screening tool, cutoff points, and identified risk strata.

Current health care and treatments for the whole cohort by risk strata and by pain site will be described using data from patient self-report questionnaires at 6 months. NHS and non-NHS resource use will be multiplied by unit costs obtained from standard sources, in order to calculate mean overall per patient costs and by broad health care-resource use type. A description of mean costs by risk strata and by pain site will be presented. Responses to the EQ-5D-5L will provide estimates of mean quality of life (utility) values, again for the whole patient group and by risk strata and pain site.

### Qualitative study

Focus groups will be conducted with patients, GPs, and other HCPs. The key aims of the focus groups for GPs and HCPs are to investigate clinicians’ perspectives on the “added value” of prognostic stratified care to current primary care management of patients who consult for musculoskeletal conditions and to identify practical implications of embedding the Keele STarT MSK tool within consultations that complement existing diagnostic approaches. For patients, the key aim is to explore their views on the most acceptable ways of communicating prognostic screening results and the understanding of a stratified model of care. For both GPs/HCPs and patients, there is also the aim of understanding any potential barriers and exploring practical solutions to the use of stratified care. If difficulty is experienced in recruiting an adequate number for the focus groups, we will conduct one-to-one interviews (face to face or over the telephone) with respondents who have expressed an interest in taking part, but have not been able to attend any of the arranged focus groups, and who agree to this alternative data-collection method.

#### Patient focus groups (and interviews)

A subsample of patients who have responded to the cohort baseline questionnaire, given consent to further contact, and provided telephone contact details will be invited to take part in a focus group. Patients will be purposively sampled to obtain a sample with diverse characteristics; including, age, socioeconomic status, and reported pain site/severity. Such information will be obtained from patients’ participation and information given in the cohort study questionnaire. We plan to convene approximately two patient focus groups (up to eight patients per group), but the final number will be determined by “data saturation” (if all themes have been explored in sufficient depth). Patients who express interest in taking part will be posted a formal invitation letter and participant information sheet. After sufficient time (approximately 2–3 days) for potential participating patients to receive and read the information and to decide whether or not to take part, patients will receive a further telephone call/s to confirm agreement and make arrangements. A date will be agreed upon, based on convenience to all patients and researchers, and a final confirmation letter of the date, time, and venue (including directions and map) will be sent to participating patients by post. Patient focus groups (and interviews) will be convened at a convenient location and setting, such as Keele University or another potential venue, such as a GP practice, other local NHS site, or the patient’s own home. Written consent will be obtained prior to the focus group discussion or one-to-one interview (as appropriate). Patients who agree to a telephone interview will receive a consent form along with their confirmation letter, and will be instructed to return it to the study team within a prepaid envelope.

#### General practitioner focus groups (and interviews)

We will invite GPs from participating practices plus GPs from known clinical networks to attend a focus group. Although no purposeful sampling will take place for the recruitment of GPs or HCPs, the study will consider a range based on experience levels and length of time in practice, as well as varying degrees of familiarity with a prognostic approach to stratified care for low-back pain. We will hold approximately four focus groups (up to eight GPs per group), though the final number will depend on data saturation. One-to-one interviews either face to face or via the telephone will be carried out as appropriate (where recruitment to focus groups has not provided an adequate sample size) and where practically possible for GPs who cannot attend a focus group. Focus groups will be held at Keele University or alternatively at one or more of the participating practices, dependent on preferences. One-to-one interviews will be conducted at a location convenient to the participating GPs, such as their practice. All GPs invited to take part will receive invitation letters and information sheets. Written consent will be obtained immediately prior to the focus-group discussion (or interview).

#### Health care professional focus groups (and interviews)

Approximately two focus groups will be convened with other HCPs (eg, such specialists as orthopedic surgeons, pain specialists, rheumatologists, and physiotherapists). However, the final number of focus groups will be dictated by data saturation. HCPs will be recruited from the services linked to participating GP practices and other known clinical networks. All HCPs will receive an invitation letter and information sheet, and written consent will be obtained immediately prior to the focus-group discussion (or interview). Focus groups (and one-to-one interviews) will be convened at either Keele University or at a convenient NHS or community venue.

#### Data collection

Each focus group will be convened in a similar way. First, a short presentation by the study team, including a summary and explanation of stratified primary care, will be delivered, followed by questions from participants in the focus groups. The presentation will include examples (vignettes) of how the Keele STarT MSK tool might be used in clinical practice and how patients might be classified into low-, medium-, and high-risk subgroups. Following the vignettes, there will be group discussion based on the topic guide, where participants will discuss each case scenario and share their views of the advantages and disadvantages, as well as the added value, face validity, acceptability, and appropriateness of stratified care for their particular pain problem (in the case of patients), and for use within clinical practice (for GPs/HCPs). One-to-one interviews will follow the same process, with a short explanation of stratified primary care, presentation of vignettes, and discussion based on the topic guide. Key emergent insights will be incorporated into the topic guides and explored in subsequent focus groups and interviews. This process is common to qualitative research, and facilitates the development of new insights not hitherto anticipated.

#### Sample size

For patients, we anticipate a 50% response rate, and so will contact approximately 32 patients, with the aim of 16 patients agreeing to take part in focus groups or interviews. We aim to recruit to four GP focus groups (up to eight per group, so a total of up to 32 GPs) and two HCP groups (up to eight per group, so a total of 16 other HCPs).

#### Study analysis

Focus-group and interview data will be transcribed and coded thematically using NVivo qualitative data-management software. Members of the research team, including social scientists and clinicians, will independently code a sample of the transcriptions and discuss their interpretations at regular meetings. Each focus group and interview will be analyzed thematically in search of similarities and differences in clinicians’ and patients’ views about the face validity, acceptability, and added value of prognostic screening linked with matched treatment options in the care of patients with musculoskeletal pain.

The qualitative data will in turn be analyzed in search of common themes using the constant comparative method,^58^ in order to identify the perceived barriers and solutions to using stratified care in clinical practice for the management of patients with the five most common musculoskeletal pain presentations. Specifically, the analysis of focus-group and interview data will be underpinned by Michie et al’s^59^ “theoretical domains framework”, previously applied to behavior change and implementation in health care settings.^60^,^61^ The framework contains 14 domains, including social, psychological, behavioral, and environmental, across which we will map the qualitative findings to determine the most salient influences on the potential for adoption of stratified primary care in practice. The analysis of the domains will help to identify the key barriers and enablers to stratified care, and identify key attributes (eg, skills, beliefs, knowledge, professional identity, environmental context), in order to address barriers or facilitate key enablers to change in future phases of the wider program of our research.

### KAPS timeline

Baseline recruitment to KAPS is planned for 6 months at each participating GP practice, each participant will then be followed up for 6 months. After completion of follow-up, final data cleaning and analysis is planned.

### KAPS data archiving and data access

The study is designed so that any participant personal data (eg, names, addresses) will be stored in a confidential, password-protected database accessible only by those with permission (eg, study team). Furthermore, all data used for analysis will be kept separate from participant personal data. Similarly, all hard-copy information (eg, signed consent forms, questionnaires) will be stored securely within the Arthritis Research UK Primary Care Centre building at Keele University. Hard-copy material will be stored for a period of 5 years after the full NIHR program has completed (5-year period for program). After that period, all hard-copy material will be destroyed. All confidentiality arrangements will adhere to relevant regulations and guidelines (Data Protection Act 1998, Caldicott review, General Medical Council, Medical Research Council, Research Governance Framework), and the chief investigator and study statistician (data custodian) will have responsibility to ensure the integrity of the data and that all confidentiality procedures are followed.

In certain circumstances, we can share access to our research databases to support joint publications and other research collaborations. Researchers wanting to apply for access to individual patient data from studies hosted by the Arthritis Research UK Primary Care Centre should complete an external data-request form and send an electronic version of the form, together with an outline design of the investigation and a short CV for all study team members external to the Arthritis Research UK Primary Care Centre. In the first instance, please contact primarycare.datasharing@keele.ac.uk.

### KAPS oversight arrangements

This study is part of a larger NIHR program of work (grant RP-PG-1211-20010), and receives input from a dedicated program steering committee (PSC). The PSC consists of an independent chair, independent research and clinical members, and also lay members (patient and public involvement). The PSC gives an independent perspective to the study and study processes, and ensures the study is conducted in accordance with research governance inclusive of adherence to protocol, procedures of consent, and patient safety.

## Discussion

This study aims to provide a validated prognostic tool (Keele STarT MSK tool), supported by information from patients and clinicians about likely acceptability and feasibility of use. The tool will enable the identification of different subgroups of patients seeking primary care for the five most common pain presentations (back, neck, shoulder, knee, multisite pain). The study will describe the patients within each risk subgroup, in terms of their clinical characteristics and health care use over a 6-month period after consultation. The tool is a starting point for a new stratified primary care model in which patients with these musculoskeletal pain presentations will be subgrouped based on prognostic risk for matched treatment. We anticipate further testing, validation, and translation of the tool from varied research groups on different musculoskeletal treatment settings (eg, interface clinics, private practice, osteopathic practice, chiropractic practice), as has been demonstrated by the STarT Back tool’s subsequent development. Further investigation of this new Keele STarT MSK tool will be required on the added value within clinical decision making, and prediction stability will require further assessment within potential subgroups and different musculoskeletal populations (eg, age, sex, cultural variation, socioeconomic status, illness duration, diagnosis category). Currently, the tool is also being used within a planned randomized cluster trial, which is investigating if stratified care, involving the use of the Keele STarT MSK tool and matched treatment options for adults who present to GPs with one of the five most common musculoskeletal pain presentations, is more clinically effective and cost-effective compared to usual unstratified care.

While this protocol describes a comprehensive plan to develop and produce a validated prognostic tool, there are some limitations. Certain compromises have been made to minimize participant burden with regard to the questionnaire at baseline and follow-up, eg, we have used single-item or shortened-version measures for such aspects as physical activity and illness perceptions, whereas full versions would yield much more informative data.

## Figures and Tables

**Table 1:** Outcome domains, measures, and timing of data collection

Domain	Measure	Questionnaire stage
Physical health	SF-36 version 2, physical component summary score^31^,^32^	All questionnaires
Pain interference	Promis Pain Interference Scale^34^	All questionnaires
Pain intensity	Pain intensity^33^	All questionnaires
Pain location	Pain location, body mannequin^43^	Baseline
Pain experience	Spread and duration of pain^33^,^44^	Baseline
Quality of life	EQ5D-5L^35^	All questionnaires
NHS and non-NHS health care utilization	Questions on primary and secondary health care contacts, investigations, treatments, medications, self-certification	2-month follow-up 6 month follow-up
Health literacy	Single-item literacy screener^45^	Baseline
Psychological reactions to pain	Coping Strategies Questionnaire (catastrophizing subscale,^46^ Pain Self-Efficacy Questionnaire, 47 selected items – revised Illness Perception Questionnaire^48^,^49^	Baseline 6-month follow-up
Sleep	Jenkins Sleep Questionnaire^50^	All questionnaires
Fatigue	SF-36 vitality scale^32^	All questionnaires
General health	SF-36 general health scale version 2^31^,^32^	All questionnaires
Mental health	SF-36 version 2 mental component summary score^31^,^32^	All questionnaires
Physical activity	Single question on physical activity^51^	All questionnaires
Change since index consultation	Global rating of change question – single item^11^,^52^	2-month follow-up 6-month follow-up
Comorbidity	Presence of other long term medical conditions	Baseline
Social support	Emotional and instrumental support^53^	Baseline
Employment	Employment status, work loss, work satisfaction	Baseline 6-month follow-up
Screening tool comparison	Örebro Musculoskeletal Pain Screening Questionnaire short form,^20^ STarT Back tool^23^	Baseline
Risk of persistent disabling pain	Draft Keele STarT MSK tool	All questionnaires
Education	Years in full-time education, further education, qualifications	Baseline
Medical record review	General practice records of consultation frequency, prescriptions, referrals, diagnostic tests, sickness/fit notes	NA
Minimal data collection	Pain intensity,^33^ SF-12,^36^ global rating of change question – single item^11^,^52^	Minimal data collection

**Abbreviations:** EQ, EuroQol; SF, short form; STarT, subgroups for targeted treatment; NA, not applicable.
